# Migration of a ventriculoperitoneal shunt to the mediastinum: A case report and literature review

**DOI:** 10.1097/MD.0000000000043465

**Published:** 2025-07-18

**Authors:** Yan Feng, Jingchen Li, Zhongjie Yan, Guozhu Sun

**Affiliations:** a Department of Neurosurgery, The Second Hospital of Hebei Medical University, Shi Jiazhuang, Hebei, China.

**Keywords:** case report, hydrocephalus, mediastinum, migration, ventriculoperitoneal shunt

## Abstract

**Rationale:**

Ventriculoperitoneal shunt (VPS) surgery, a common neurosurgical procedure for hydrocephalus, carries risks of complications such as shunt obstruction and infection. Migration of the shunt into the mediastinum, however, is an uncommon complication, particularly in adults.

**Patient concerns:**

A 56-year-old male patient was admitted to our hospital with a complaint of “progressive decline in mobility over 3 months, significantly impairing daily activities.” He had previously undergone bilateral VPS surgery and was admitted with a suspected shunt obstruction.

**Diagnoses:**

Chest radiography showed that the left ventricular-ventral end shunt had migrated through the supra-aortic arch and mediastinum to the right atrial region. Chest computed tomography showed that the left shunt image entered the mediastinum, and multiple strip high-density images could be seen in the mediastinal cavity.

**Interventions:**

External drainage of a right VPS catheter was performed. Intraoperative findings revealed that the left-sided peritoneal catheter migrated into the thoracic cavity. Nineteen days later, the bilateral VPS catheters were revised, and the left shunt catheter was extracted from the thoracic cavity and repositioned into the abdominal cavity; the right shunt catheter was reinserted into the peritoneal cavity.

**Outcomes:**

Both shunt catheters were successfully repositioned into the peritoneal cavity. No thoracic or abdominal complications were documented during the perioperative period. During the 6-month follow-up period, the patient maintained neurological improvement without complication recurrence.

**Lessons:**

After VPS surgery, shunt migration should be considered a potential cause of obstruction. Once identified, the migrated shunt tube should be promptly readjusted to restore its proper function.

## 1. Introduction

A ventriculoperitoneal shunt (VPS) is a classic surgical procedure for treating hydrocephalus. It involves implanting a shunt catheter to divert cerebrospinal fluid (CSF) from the cerebral ventricles to the peritoneal cavity, thereby alleviating elevated intracranial pressure.^[1]^ Indications for VPS include various types of hydrocephalus, such as obstructive hydrocephalus, communicating hydrocephalus, and normal-pressure hydrocephalus.^[2]^ Despite significant advancements in surgical techniques and shunt device design, the failure rate of VPS devices remains as high as 11 to 25% within the first year post-implantation. Over a 20-year period following shunt placement, patients undergo an average of 2 to 3 surgical revisions for shunt adjustment or replacement.^[3]^ Malfunction of the VPS system can significantly increase disability and mortality rates, necessitating rapid and accurate diagnosis, as well as timely repair.^[4]^ Common postoperative complications of VPS surgery include shunt blockage, obstruction, infection, migration, subdural hematomas, etc.^[5,6]^ Among these, shunt migration is an uncommon complication, most commonly observed in children.^[7]^ The literature indicates that displaced shunts can migrate to various locations, including the stomach, chest, lungs, bladder, anus, and heart.^[8–11]^ While the literature indicates that shunt migration occurs more frequently in pediatric patients, adults may also experience this complication due to distinct mechanisms such as tissue laxity, intra-abdominal pressure fluctuations, or comorbidities.^[12]^ It is critical to emphasize that adult patients often present with atypical symptoms (e.g., subtle cognitive decline, intermittent headaches, respiratory distress, and neurological decline), leading to delayed diagnosis. This delay increases the risk of severe secondary complications, including intracranial hypertension and brain herniation.^[8,13]^ Consequently, shunt migration represents a potentially serious complication of VPS surgery and warrants particular vigilance in the adult population. We report a case of migration of a VPS to the mediastinum in an adult patient. The patient complained that “the mobility decreased progressively for 3 months, seriously affecting daily activities.” The patient had undergone left VPS surgery 5 months previously. Brain computed tomography (CT) revealed hydrocephalus, suspected to be caused by shunt obstruction. Chest radiography and CT further showed that the left VPS had migrated into the right thoracic cavity. The patient underwent reoperation to adjust the shunt, resulting in good recovery.

## 2. Case presentation

### 2.1. Patient information

A 56-year-old male patient was admitted to the Neurosurgery Department of the Second Hospital of Hebei Medical University on June 21, 2023, with the chief complaint of “progressive decline in mobility over 3 months, significantly impairing daily activities.” His medical history included hypertension for 30 years, which was well-controlled with oral enalapril and amlodipine besylate. Seven years ago, due to a “right basal ganglia hemorrhage,” he underwent craniotomy to remove a cerebral hemorrhage hematoma, as well as a decompressive craniotomy at our hospital. Six years ago, he underwent cranial defect repair and right VPS placement for a right frontotemporal-parietal skull defect and hydrocephalus at our hospital. Five months prior, the patient underwent left VPS surgery at our hospital for hydrocephalus.

### 2.2. Clinical findings

**Figure 1.**
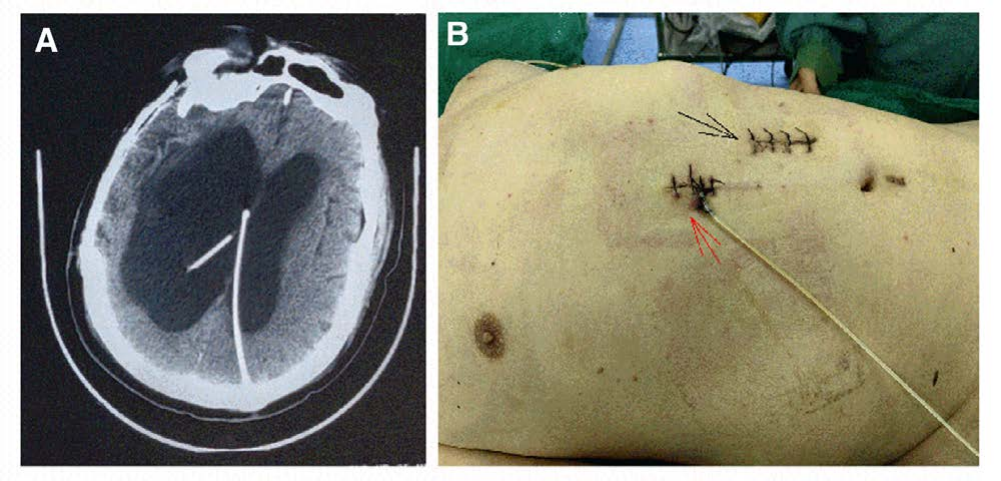
(A) Brain CT showing hydrocephalus with changes observed after bilateral ventricular shunt placement. (B) After adjustment of the peritoneal end of the shunt tube in both ventricles, the shunt on the left side of the abdomen was not explored (black arrows), and the right ventricular drain was externally drained (red arrows). CT = computed tomography.

Three months prior, the patient presented with progressive mobility impairment, manifested as an inability to walk and postural instability. This is accompanied by slowed responsiveness, urinary incontinence, and cognitive decline. Vital signs: T 36.6°C, P 68 bpm; R, 20 breaths/min; and BP, 116/90 mm Hg. A physical examination revealed lethargy and slurred speech. The bilateral pupils measured 2.5 mm with intact light reflexes. There was no neck stiffness, and muscle strength was grade II in the left limbs and grade IV in the right limbs. Normal deep/superficial reflexes were observed bilaterally, and the bilateral Babinski signs were negative. A brain CT scan (Fig. 1A) showed postoperative changes from right frontotemporoparietal skull repair, soft foci in the right frontotemporoparietal region, and hydrocephalus with evidence of bilateral VPS surgery.

### 2.3. Timeline of the current episode

**Figure 2.**
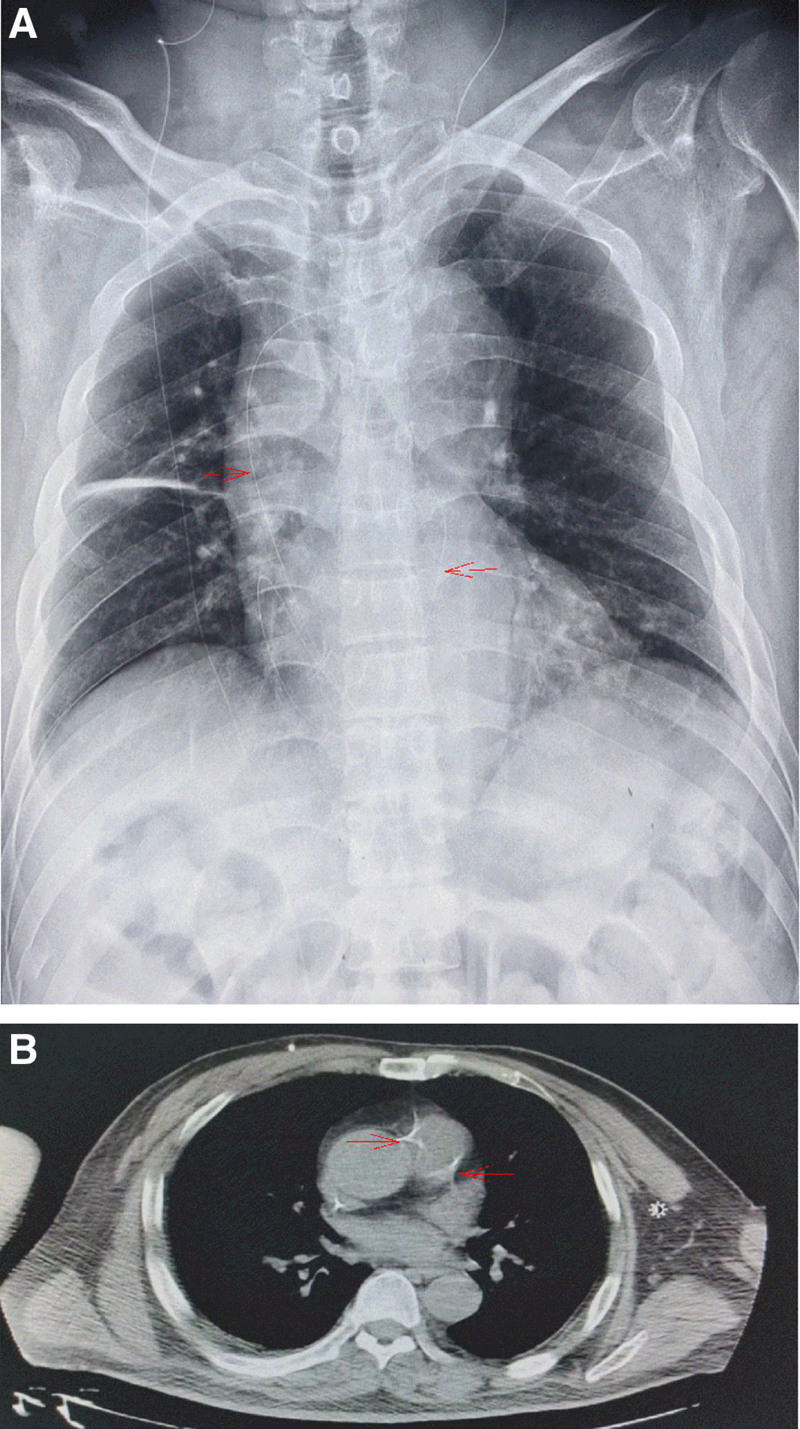
(A) Chest radiograph showing the left ventral end of the shunt coiled in the thoracic cavity (indicated by the red arrow). (B) Chest CT showing migration of the left ventral end shunt into the mediastinal cavity (indicated by the red arrow).

**Figure 3.**
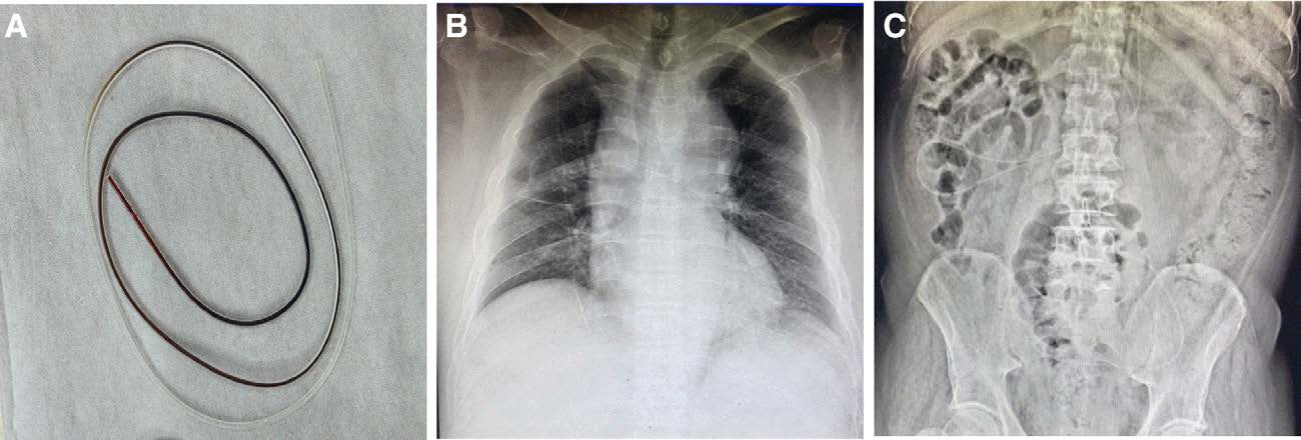
(A) After removal of the mediastinal luminal shunt, a thrombus was visible inside the shunt. (B and C) Postoperative chest and abdominal radiographs showing proper positioning of the abdominal end of the shunt bilaterally. CT = computed tomography.

The patient was admitted to the Department of Neurosurgery of our hospital on June 21, 2023. The peritoneal ends of the 2 shunt catheters were suspected to be occluded. Upon hospital admission, a laboratory workup was completed, but no preoperative chest radiologic studies were performed. On June 25, 2023, the patient underwent external drainage of a right ventricular-peritoneal shunt catheter under general anesthesia. The skin and subcutaneous tissue of the abdomen were cut along the original right abdominal incision under the xiphoid process, and the shunt tube was pulled out of the abdominal cavity, revealing that the holes and ports at the end of the shunt tube were obstructed. The end of the shunt tube was cut off for about 5cm. The remaining shunt tube was confirmed to be smooth, and the CSF appeared light yellow. The drainage tube at the abdominal end was temporarily placed outside the body for extraperitoneal drainage (Fig. 1B). Abdominal skin and subcutaneous tissue were incised along the original left abdominal incision. Unfortunately, this shunt has not yet been explored. An immediate intraoperative chest radiograph (Fig. 2A) showed that the left ventricular-ventral end shunt had migrated through the supra-aortic arch and mediastinum to the right atrial region. The surgical procedure was temporarily terminated after considering the migration of the left shunt into the thoracic cavity. On June 26, 2023, a chest CT scan (Fig. 2B) showed that the left shunt image could be seen entering the mediastinum, and multiple strip high-density images could be seen in the mediastinal cavity. After 19 days of continuous external drainage from the right ventricle to the abdominal end, the patient’s mental status significantly improved. He was alert, clear-minded, and able to respond simply and correctly. He was able to sit up with assistance from his family, and CSF laboratory test results were normal. On July 14, 2023, under general anesthesia, the right ventricular-abdominal end drain was repositioned into the abdominal cavity. A left occipital incision was made to expose the ventral end port of the left shunt pump, and the shunt tube was removed from the mediastinal cavity where thrombus occlusion was observed at the ventral end of the tube (Fig. 3A). A new subcutaneous tunnel was established and the peritoneal drainage tube was replaced and placed into the abdominal cavity. Postoperative chest and abdominal radiography showed that the abdominal ends of both shunts were well-positioned (Fig. 3B and C), and the patient was successfully discharged.

### 2.4. Diagnostic assessment

Chest radiography (Fig. 2A) showed that the left ventricular-ventral end shunt migrated through the supra-aortic arch and mediastinum to the right atrial region. A chest CT scan (Fig. 2B) showed that the left shunt image entered the mediastinum, and multiple strip high-density images could be seen in the mediastinal cavity.

### 2.5. Diagnosis

Diagnosis: hydrocephalus and VPS tube disorder (shunt migration). Basis for diagnosis: age: 56 years, male; chief complaint: progressive decline in mobility over 3 months, significantly impairing daily activities; past medical history: underwent bilateral ventriculoperitoneal shunt surgery; clinical presentation: consciousness: lethargic; pupils: bilateral pupil size 2.5 mm, intact light reflex; neck: no stiffness; limb strength: left limbs: Grade II muscle strength; right limbs: Grade IV muscle strength; and chest CT findings: displaced shunt catheter observed in the mediastinum.

### 2.6. Therapeutic interventions

On June 25, 2023, external drainage of the right ventriculoperitoneal (VP) catheter was performed. Exploration of left diversion pipe failure. Intraoperative chest radiograph (Fig. 2A): the left catheter was displaced to the right atrial area via the mediastinum above the aortic arch. On July 14, 2023, a right external drainage tube was inserted into the abdominal cavity. The faulty catheter on the left side was removed, and a new catheter was inserted into the abdominal cavity.

### 2.7. Follow-up and outcomes

Both shunt catheters were successfully repositioned into the peritoneal cavity. Postoperative chest and abdominal radiographs confirmed appropriate bilateral catheter positioning within the peritoneal cavity. No thoracic or abdominal complications were documented during the perioperative period. During the 6-month follow-up period, the patient maintained neurological improvement without complication recurrence.

## 3. Discussion

Complications of shunt surgery can occur along the entire course of the shunt from the ventricles to the abdominal cavity. Intrathoracic displacement of the distal shunt is a rare postoperative complication that can lead to serious complications such as pleural effusion, pneumonia, fever, dyspnea, and shunt obstruction. In the international literature, cases of thoracic displacement in adults are scarcer; most reports focus on the child group,^[14–19]^ and most patients are treated for clinical manifestations such as pleural effusion or pneumonia. We report a case of distal displacement of the shunt to the thoracic cavity in an adult patient with no related thoracic symptoms. The patient was admitted to the hospital because of recurrence of hydrocephalus symptoms caused by shunt obstruction. Notably, during our first abdominal shunt placement for external drainage, we did not anticipate left-sided shunt displacement into the thoracic cavity. Therefore, for patients with shunt dysfunction following VPS placement, the possibility of shunt displacement should be considered even in the absence of thoracic symptoms (such as cough and expectoration, pulmonary inflammation, and pleural effusion, etc). Comprehensive preoperative laboratory tests and chest radiography are necessary.

Although cases of shunt migration into the thoracic cavity are relatively rare, previous reports have documented these occurrences. For instance, Lyon et al^[20]^ described an elderly male patient whose primary symptoms manifested as worsening of motor and cognitive functions. Three weeks after the VPS surgery, the patient’s symptoms recurred. Chest CT showed that the distal shunt had migrated to the heart and pulmonary vessels. The retroauricular neck incision was reopened, and the distal catheter was carefully withdrawn. They elected not to revise their shunt during the same sitting and placed him under brief observation to rule out any pulmonary or cardiac injury. Two months later, he underwent distal revision of the contralateral side of the neck. The above-reported cases are similar to ours, as the patients in both instances did not experience pneumonia or symptoms of chest discomfort. Unlike these cases, during our procedure, we extracted the catheter from the left mediastinum and reestablished a new subcutaneous tunnel before placing a new catheter into the abdominal cavity. Liu et al^[21]^ reported the case of a 56-year-old male who underwent VP shunt surgery for hydrocephalus after aneurysm surgery. Nine years later, the patient developed general weakness and abdominal pain. Chest radiography revealed that the peritoneal end of the shunt tube moved to the pleural cavity with the formation of pulmonary inflammation. The peritoneal end of the shunt tube was removed, and a VPS was re-performed after anti-inflammatory treatment. Unlike our case, this patient experienced a later onset of catheter migration and developed severe pulmonary inflammation.

Various theories have been proposed regarding the mechanism by which the shunts migrate to the thorax. Taub et al classified distal migration of the shunt into the thoracic cavity after VPS surgery into 2 types: supradiaphragmatic migration and transdiaphragmatic migration.^[22]^ According to anatomical definitions, the superior mediastinum is the region of the superior thoracic aperture, which is the entrance to the thoracic cavity and is located between the upper edge of the sternal manubrium and top of the first rib. Supradiaphragmatic catheter migration into the thoracic cavity is primarily attributed to unintentional injury to this area during the creation of the subcutaneous tunnel in the supraclavicular fossa and rib regions during surgery.^[14,15]^ Over time, negative inspiratory pressure or increased patient motion may cause the shunt catheter to gradually herniate into the thoracic cavity through this breach, until it is fully displaced. Transdiaphragmatic distal catheter migration into the thoracic cavity is thought to result from erosion of the diaphragm by the shunt.^[22,23]^ For example, inflammation may lead to erosive perforation of the diaphragm, allowing the abdominal shunt to migrate into the thoracic cavity, or displacement may occur through congenital fissures and weak areas in the diaphragm, such as thoracoabdominal hiatal hernias (Bochdalek foramen) or paraspinal hernias (Morgagni foramen). Consequently, the distal end of the shunt may migrate from the abdominal cavity through the diaphragm into the thoracic cavity or, in rare cases, into the lungs.

We reviewed the relevant reports on shunt tube migration in the chest and lungs (Table 1). As shown in Table 1, most patients were treated for clinical manifestations, such as pleural effusion or pneumonia,^[14–19]^ and most of them were children or minors. Most patients had a favorable prognosis after prompt shunt removal or repositioning. The most common mechanism of shunt migration is transseptal displacement into the thoracic cavity,^[16–19,22]^ followed by displacement into the thoracic cavity due to incorrect subcutaneous tunneling.^[15]^ However, in our case, the chest CT scan and intraoperative manipulation showed that the distal displacement of the shunt into the thoracic cavity might be related to an incorrectly created subcutaneous tunnel at the upper thoracic opening during the first surgical procedure. The clinical manifestation in the patient was hydrocephalus. Upon removal of the shunt tube from the abdominal cavity, a thrombus was found in the shunt tube, which was blocked, consistent with the clinical manifestation of the patient. No signs of pneumonia or pleural effusion were observed. We analyzed the possible causes as follows: the shunt tube entering the pleural cavity was blocked, preventing fluid from being shunted into the pleural cavity; the shunt in the thoracic cavity did not induce an inflammatory reaction, preventing further movement into the pulmonary artery or lung tissue; and the patient’s symptoms appeared early, and the condition was detected and treated in a timely manner.

**Table 1:** Reported cases of supradiaphragmatic intrathoracic migration.

Cases	Age	The duration between shunt placement and migration	The location of migration	Radiologic findings	Mechanism	Surgery	Outcome
Obrador and Villarejo, 1977^[15]^	17 months	13 months	Inside the thoracic cavity	Chest X-ray showed pleural effusion	Incorrect subcutaneous passage	Reposition in the peritoneal cavity	Recovery
Cooper, 1978^[16]^	7 months	21 d	The right thoracic cavity	Chest X-ray showed pleural effusion	Xiphocostal margin beneath rectus abdominis muscle	Shunt removal + Distal catheter replacement	Recovery
Anegawa et al, 1986^[17]^	23 yr old	4 yr	The right hilar area with local pneumonitis	Chest X-ray showed pneumonitis	Transdiaphragmatic	Shunt removal + Distal catheter replacement	Recovery
Taub et al 1994^[23]^	42 yr old	6 yr	The bronchus of the right chest	Chest X-ray showed bronchial shadows by injection of iodohexanol solution	Transdiaphragmatic	Distal catheter replacement	Recovery
Sahin et al 2007^[18]^	15 yr old	13 yr	The right lung parenchyma	Chest X-ray and CT showed pneumonia	Transdiaphragmatic	Distal catheter replacement	Recovery
Nazaroğlu et al 2009^[19]^	45 yr old	3 yr	migrated into the right lung by a transdiaphragmatic and transhepatic route	Chest CT showed pneumonia + hydrothorax	Transdiaphragmatic	Shunt removal	Recovery
Lyon K et al 2016^[21]^	71 yr old	3 wk	The pulmonary artery	Chest CT showed catheter migration into the pulmonary vasculature	Transdiaphragmatic	Shunt removal + Distal catheter replacement	Recovery
Liu et al 2020^[22]^	56 yr	9 yr	The pleural cavity	Chest X-ray showed pneumonia + hydrothorax	Transdiaphragmatic	Shunt removal + Distal catheter replacement	Recover
Ruiz Johnson A, 2021^[20]^	10 yr old	10 yr	The right pleural effusion	Chest X-ray and CT showed pneumonia + pleural effusion	Transdiaphragmatic	Distal catheter replacement	Recovery

CT = computed tomography.

To prevent the migration of the VPS into the thoracic cavity and lungs, the following precautions should be taken: the shunt should be soft, the abdominal end should not be too long, a length of 20 to 30 cm is appropriate, and the end of the shunt should be round and blunt. When creating a subcutaneous tunnel through the neck and chest area, the shunt should be positioned precisely under the skin, neither too deep nor too shallow. If placed too deep, the internal jugular vein could be punctured, or the thoracic cavity could be injured; if placed too shallow, the skin may be penetrated. The subcutaneous tunnel in the cervicothoracic region should be properly accessible, and the shunt should be positioned correctly after the tunnel strip is removed; the site for the abdominal shunt placement should be away from previous surgical incisions; regular brain CT scans and chest and abdominal X-rays should be conducted after VPS surgery to monitor the location of the shunt; and attention should be paid to any improvement in hydrocephalus symptoms of the patient after the shunt surgery, as well as to early symptoms of abnormal chest discomfort, such as fever, cough, and mild respiratory distress, to ensure timely checkups and treatments.

Certainly, our study has inherent limitations. As a single-center case report, while we analyzed and synthesized the causes of shunt migration in the discussion section, it lacks multi-center validation and large-scale sample data. Future efforts should focus on establishing multi-center databases to identify risk factors for mediastinal VPS migration in adult patients, such as variations in surgical techniques, catheter material properties, and intrathoracic pressure dynamics. VPS surgery is commonly used for the treatment of hydrocephalus, and strict, continuous postoperative follow-up is an effective means of detecting shunt obstruction. A careful preoperative search for the cause of shunt obstruction is essential because shunt migration can also contribute to obstruction. Although shunt migration into the thorax and lungs is extremely rare, it can cause serious complications. Therefore, scholars emphasize that thoracic migration of the VPS constitutes an insidious neurosurgical emergency that requires urgent intervention. The absence of thoracic symptoms does not exclude the possibility of displacement; in evaluating shunt dysfunction, thoracic imaging (radiography or CT) must be included without compromise. Once clinically identified, the migrated shunt must be realigned.

## 4. Conclusion

Thorough preoperative examination, standardized intraoperative procedures, and strict postoperative review are important for reducing complications after VPS surgery. Although shunt migration is relatively rare, it can lead to serious complications. Therefore, shunt migration should be considered in patients with shunt dysfunction after VPS. Prompt adjustment of the migrated shunt is essential for avoiding serious adverse consequences.

## Author contributions

**Data curation:** Yan Feng.

**Project administration:** Jingchen Li.

**Writing – original draft:** Yan Feng.

**Writing – review & editing:** Zhongjie Yan, Guozhu Sun.
